# Up-regulation of CLDN1 in gastric cancer is correlated with reduced survival

**DOI:** 10.1186/1471-2407-13-586

**Published:** 2013-12-10

**Authors:** Lars L Eftang, Ying Esbensen, Tone M Tannæs, Gustav P Blom, Ida RK Bukholm, Geir Bukholm

**Affiliations:** 1 Department of Clinical Molecular Biology and Laboratory Sciences (EpiGen), Division of Medicine, Akershus University Hospital and University of Oslo, N-1478 Nordbyhagen, Oslo, Norway; 2 Department of Gastrointestinal Surgery, Akershus University Hospital, Lørenskog, Norway; 3 Department of Pathology, Akershus University Hospital, Lørenskog, Norway; 4 Department of Breast and Endocrine Surgery, Akershus University Hospital, Lørenskog, Norway; 5 Institute of Clinical Medicine, Akershus University Hospital and University of Oslo, Lørenskog, Norway; 6 Department of Infection Prevention, Oslo University Hospital, Oslo, Norway; 7 Department of Chemistry, Biotechnology and Food Science, Norwegian University of Life Sciences, Ås, Norway

**Keywords:** Gastric cancer, Interlukin 8, Claudin-1, *Helicobacter pylori*, cDNA microarray, Survival, Prognosis

## Abstract

**Background:**

The genetic changes in gastric adenocarcinoma are extremely complex and reliable tumor markers have not yet been identified. There are also remarkable geographical differences in the distribution of this disease. Our aim was to identify the most differentially regulated genes in 20 gastric adenocarcinomas from a Norwegian selection, compared to matched normal mucosa, and we have related our findings to prognosis, survival and chronic *Helicobacter pylori* infection.

**Methods:**

Biopsies from gastric adenocarcinomas and adjacent normal gastric mucosa were obtained from 20 patients immediately following surgical resection of the tumor. Whole genome, cDNA microarray analysis was performed on the RNA isolated from the sample pairs to compare the gene expression profiles between the tumor against matched mucosa. The samples were microscopically examined to classify gastritis. The presence of *H. pylori* was examined using microscopy and immunohistochemistry.

**Results:**

130 genes showed differential regulation above a predefined cut-off level. Interleukin-8 (*IL-8*) and Claudin-1 (*CLDN1*) were the most consistently up-regulated genes in the tumors. Very high *CLDN1* expression in the tumor was identified as an independent and significant predictor gene of reduced post-operative survival. There were distinctly different expression profiles between the tumor group and the control mucosa group, and the histological subsets of mixed type, diffuse type and intestinal type cancer demonstrated further sub-clustering. Up-regulated genes were mapped to cell-adhesion, collagen-related processes and angiogenesis, whereas normal intestinal functions such as digestion and excretion were associated with down-regulated genes. We relate the current findings to our previous study on the gene response of gastric epithelial cells to *H. pylori* infection.

**Conclusions:**

*CLDN1* was highly up-regulated in gastric cancer, and *CLDN1* expression was independently associated with a poor post-operative prognosis, and may have important prognostic value. *IL-8* and *CLDN1* may represent central links between the gene response seen in acute *H. pylori* infection of gastric epithelial cells, and ultimately gastric cancer.

## Background

Gastric cancer (GC) is only second to lung cancer in world-wide cancer-related deaths, however there are great geographical differences in GC distribution. Data from 2010 demonstrate that the GC incidence in Norway is very low (males 6.9, females 3.0 per 100.000) [1] compared to less developed areas, particularly in Eastern Asia, where the incidence is approximately 6-fold (males 42.4, females 18.3 per 100.000) [2].

Gastric adenocarcinoma is remarkably heterogeneous genetically, cytologically and architecturally compared to other gastrointestinal carcinomas. The search for reliable tumor markers and consistent prognostic indicators has proven difficult. Several authors have attempted to predict GC disease and prognosis based on single or multiple genes [3-8], but there are discrepancies between the studies, and currently no gene signature or biomarkers are in routine clinical use. Understanding the mechanisms underlying gastric cancer is one of the major challenges in cancer genomics. The Lauren classification divides adenocarcinomas into three different histological subtypes: intestinal and diffuse types and a mixed variant [9], which are thought to take different pathways of carcinogenesis. The intestinal type is attributable to a multistep progression from chronic gastritis through gastric atrophy, metaplasia, dysplasia and ultimately malignant disease [10]. Diffuse types may arise from chronic inflammation *without* a clear manifestation of intermediate premalignant steps [11-13]. The mixed type shows non-homogenous mixtures of both intestinal and diffuse type architecture, and might represent a separate cancer category with exclusive gene mutations and a more aggressive course [14,15]. In spite of extensive research into the genetic changes of GC, the mechanisms underlying the disease are still far from understood, and the disease cannot easily be explained by an adenoma-carcinoma model like in colorectal cancer. There are three molecular mechanisms that drive gastric carcinogenesis: Chromosome instability, microsatellite instability and epigenetic alterations [16]. The net result is activation of oncogenes, inactivation of tumor suppressor genes and deregulation of signaling pathways [11,12]. Aberrant cell cycle regulation and changes in the expression of growth factors and cytokines regulate differentiation and survival of tumor cells. Mutations of cell-adhesion and angiogenic genes play important roles in the invasive and metastatic behavior of GC cells.

The aim of the current study was to identify the most differentially regulated genes in surgically resected gastric adenocarcinoma compared to matched normal mucosa, using whole genome cDNA microarray profiling. We also attempt to identify genes which influence GC prognosis and survival. The results are compared to the gastric epithelial cell gene response to *H. pylori* infection, which was analyzed in a previously published paper [17]. This study adds support to the significance of *IL-8* and *CLDN1* in gastric carcinogenesis, as well as demonstrates important genetic changes in GC and their possible relevance to *H. pylori* infection.

## Methods

### Tissue and patient characteristics

Biopsies were obtained from patients diagnosed with non-cardia gastric adenocarcinoma at the endoscopy outpatient clinic at Akershus University Hospital, Norway. Thoraco-abdominal computed tomography imaging was undertaken to exclude patients with metastatic disease. 20 patients with both intestinal and diffuse types of GC were included. Patients and clinicopathological characteristics are presented in Table 1. On admission for elective surgery, written, informed consent for participation in the study was obtained from the participants. Within 5 minutes of removal of the principal surgical specimen, samples were taken from both the tumor border and from healthy gastric corpal mucosa within the same stomach area but more than 5 cm away from the tumor, and stored on RNA*later* (Applied Biosystems, USA). All samples were stored in +4°C for approximately 1–2 weeks to allow complete tissue penetration of RNA*later*, before samples were dried and permanently stored in -80°C. All sample acquisition and handling were performed by the same individual.

**Table 1:** Patient characteristics and clinicopathological features of the 20 gastric tumors used in the study

Sex		Females n = 5, males n = 15
Ethnicity		Caucasian n = 18, Asian n = 2
Age at surgery		Total: 68.7 years (±12.5)
		Females: 65.7 years (±21.8)
		Males: 69.7 years (±8.6)
Postoperative survival (deceased individuals)		Total: 13.2 months (±8.8)
		Females (n = 4): 16.6 months (±6.4)
		Males (n = 10): 12.0 months (±9.7)
Postoperative survival (alive individuals at study end)		Total: 45.8 months (±7.9)
		Females (n = 1): 48.0 months
		Males (n = 5): 44.9 months (±8.8)
Tumor size		49 mm (±27)
Tumor stage	T1	2
	T2	10
	T3	5
	T4	3
Nodal stage	N0	10
	N1	5
	N2	3
	N3	2
Histological type	Intestinal	5
	Diffuse	12
	Mixed	3

Patient characteristics and clinicopathological features of the 20 gastric tumors used in the study. Values are the mean plus/minus standard deviation.

Following resection of the tumor, the principal specimen was subjected to histolopathological examination by two senior specialist pathologists to confirm the diagnosis and classify the tumor according to the Lauren classification [9]. Antral and corpal gastric mucosa were examined for gastritis, atrophy and metaplasia, and the presence or absence of *H. pylori* was microscopically examined and subsequently identified by immunohistochemistry. The Updated Sydney System was used to classify and grade the degree of gastritis [18,19].

The study was approved by the Norwegian Regional Committee for Medical and Health Research Ethics (REC South East). All samples and patient data was coded and blinded before analysis.

### RNA isolation, quality control and cDNA synthesis

Total RNA was isolated using the RNeasy Blood and tissue kit (Qiagen GmBH, Germany) according to the manufacturer’s standard preparation protocol. RNA concentration and quality were determined using a NanoDrop ND-1000 spectrophotometer (NanoDrop Technologies, USA) and Agilent 2100 Bioanalyzer (Agilent Technologies, USA). The RNA integrity number was adequate for cDNA synthesis.

The Illumina TotalPrep RNA amplification Kit (Ambion Inc., USA) was used to amplify RNA for hybridization on Illumina BeadChips. To synthesize first strand cDNA by reverse transcription, we used total RNA from each sample collected above. Following the second strand cDNA synthesis and cDNA purification steps, the *in vitro* transcription to synthesize cRNA was prepared overnight for 12 hours.

### cDNA oligonucleotide microarray analysis

The gene expression profiles were measured using Illumina Human HT-12 v3 Expression BeadChip (Illumina, USA), which enables genome-wide expression analysis (48800 transcripts, corresponding to approximately 37800 genes) of 12 samples in parallel on a single microarray. 35967 of the probes were designed using the RefSeq (build 36.2, release 22) library and 12.837 probes were derived from the UniGene (build 199) database [20,21].

### Immunohistochemistry

The presence of *H. pylori* in the surgical specimens was analyzed using a polyclonal anti-*Helicobacter*-antibody (Dako, Denmark, code B0471, dilution 1:200). 4 μm sections of formalin-fixed, paraffin-embedded tissue from non-tumorous mucosa were applied on coated slides. Deparaffinization, rehydration and epitope retrieval were performed in a Dako PT Link (Dako, Denmark) at 97°C for 20 min. The immunostaining procedure was carried out in a Dako Autostainer Plus applying the Envision™Flex, High pH system (Dako, Denmark).

### Bioinformatics and statistics

R/BioConductor [22,23] with the package Beadarray [24] were used for preprocessing of the microarray text data from BeadStudio. Spatial artifacts were removed using BASH [25] before the expression data were log_2_-transformed and quantile normalized. The log_2_ fold change (FC) of each probe on the array within each tissue pair (tumor vs matched normal mucosa) was then calculated, and the data were loaded into the J-express software package [26]. Rank product testing [27] was then performed to test whether the differential expression between tumor tissue and matched normal mucosa was significant. The differential expression was declared significant if the adjusted p-value, i.e. the FDR q-value, was less than 0.05. Hierarchical clustering was performed using average linkage and Euclidean distance measure. The analyses were performed using the J-express software package [26].

To produce a reasonably sized list of the most differentially expressed genes, lesser expressed genes were filtered out at a cutoff level of FC > 1.5, producing a list of the 130 most differentially expressed genes. This dataset was imported into Onto-Express and Pathway Express [28,29], part of the Onto-Tools software suite, for functional analysis, and grouped into Gene Ontology (GO) terms and KEGG (Kyoto Encyclopedia of Genes and Genomes) cellular signaling pathways [30]. Pathway Express calculates an Impact Factor (IF) which is used to rank the affected signaling pathways, based on the fold change, the number of the involved genes in the pathway, and the amount of perturbation of downstream genes [31].

The dataset was entered into PASW Statistics (SPSS version 18.0.2) to perform bivariate correlation analysis to select genes that associated with clinicopathological parameters. Both Pearson and Spearman correlation coefficients were employed to identify correlating genes. Among genes that correlated, we were particularly interested in those that showed a similar expression in our previously published study of *H. pylori*-exposed gastric epithelial cells [17]. The selected genes were then subjected to a Cox multivariate regression analysis to investigate whether any of the genes were independent predictors of post-operative survival in the GC patients, independent of histological type, tumor stage and size, nodal disease, and age at surgery. In the one predictor gene that was identified, different cut-off levels were applied to construct high and low expression level groups, before statistical significance between the groups was assessed using a log-rank (Mantel-Cox) test. A Kaplan-Meier survival plot was created to demonstrate the difference in survival between the high- and low-expression groups.

The microarray data are available under the accession number E-MTAB-1440 in the ArrayExpress database [32].

## Results

### Gene expression

**Figure 1:**
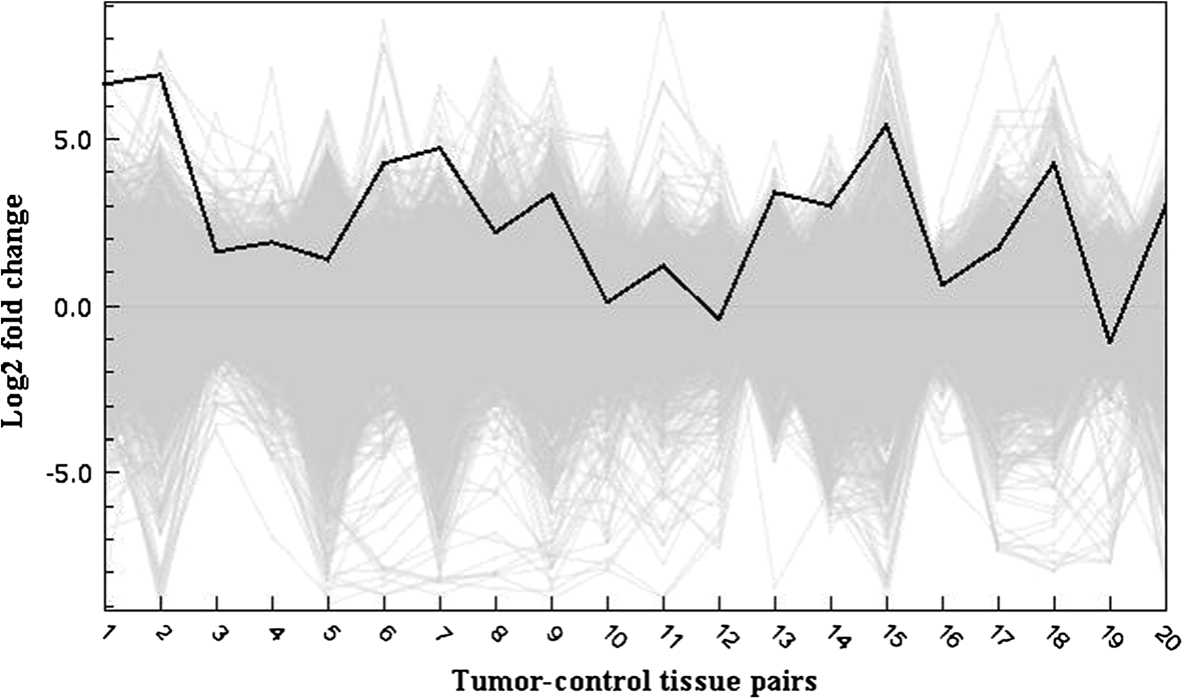
**Interleukin-8 gene expression in gastric tumors vs matched control mucosa.** The solid line represents the relative ratio of *IL-8* expression in tumor tissue compared to matched control gastric mucosa, as the log_2_ fold change (log_2_ tumor/control expression levels). A positive score indicates a higher expression in the tumor compared to the normal gastric mucosa. *IL-8* was the most consistently up-regulated gene in the study. The grey background represents the expression of approximately 37 800 other genes.

**Figure 2:**
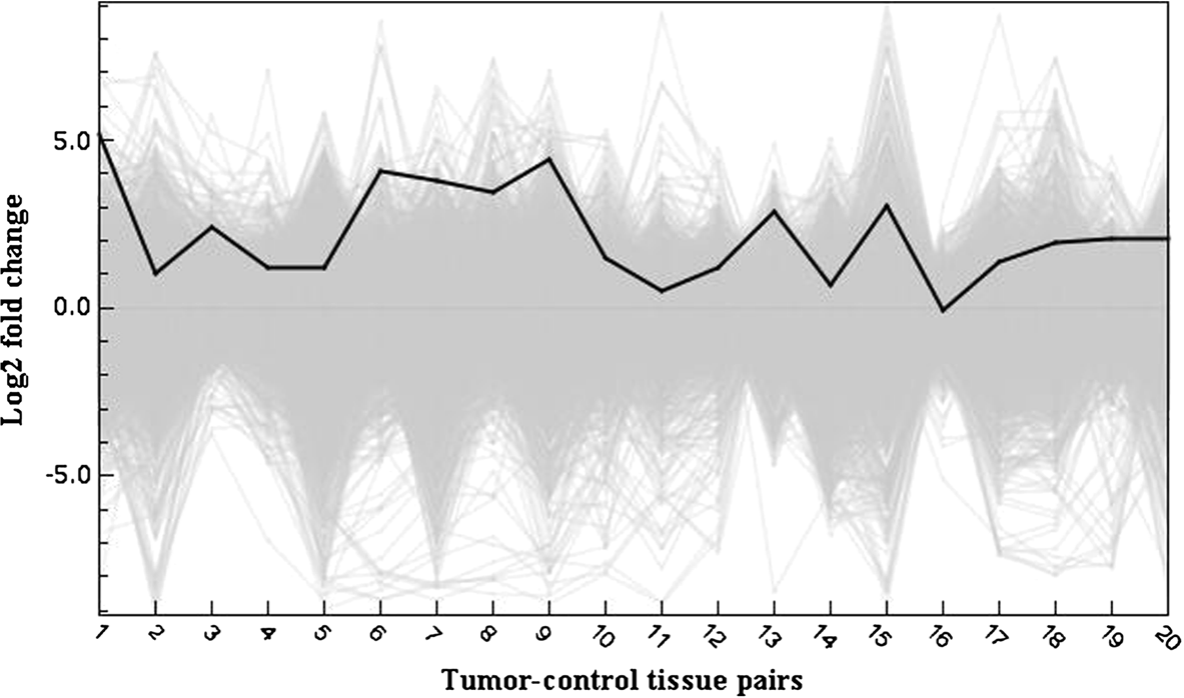
**Claudin 1 gene expression in gastric tumors vs matched control mucosa.** The solid line represents relative ratio of *CLDN1* expression in the tumor tissue compared to matched control gastric mucosa, as the log_2_ fold change (log_2_ tumor/control expression levels). A positive score indicates a higher expression in the tumor compared to the normal gastric mucosa. The grey background represents the expression of approximately 37 800 other genes.

**Figure 3:**
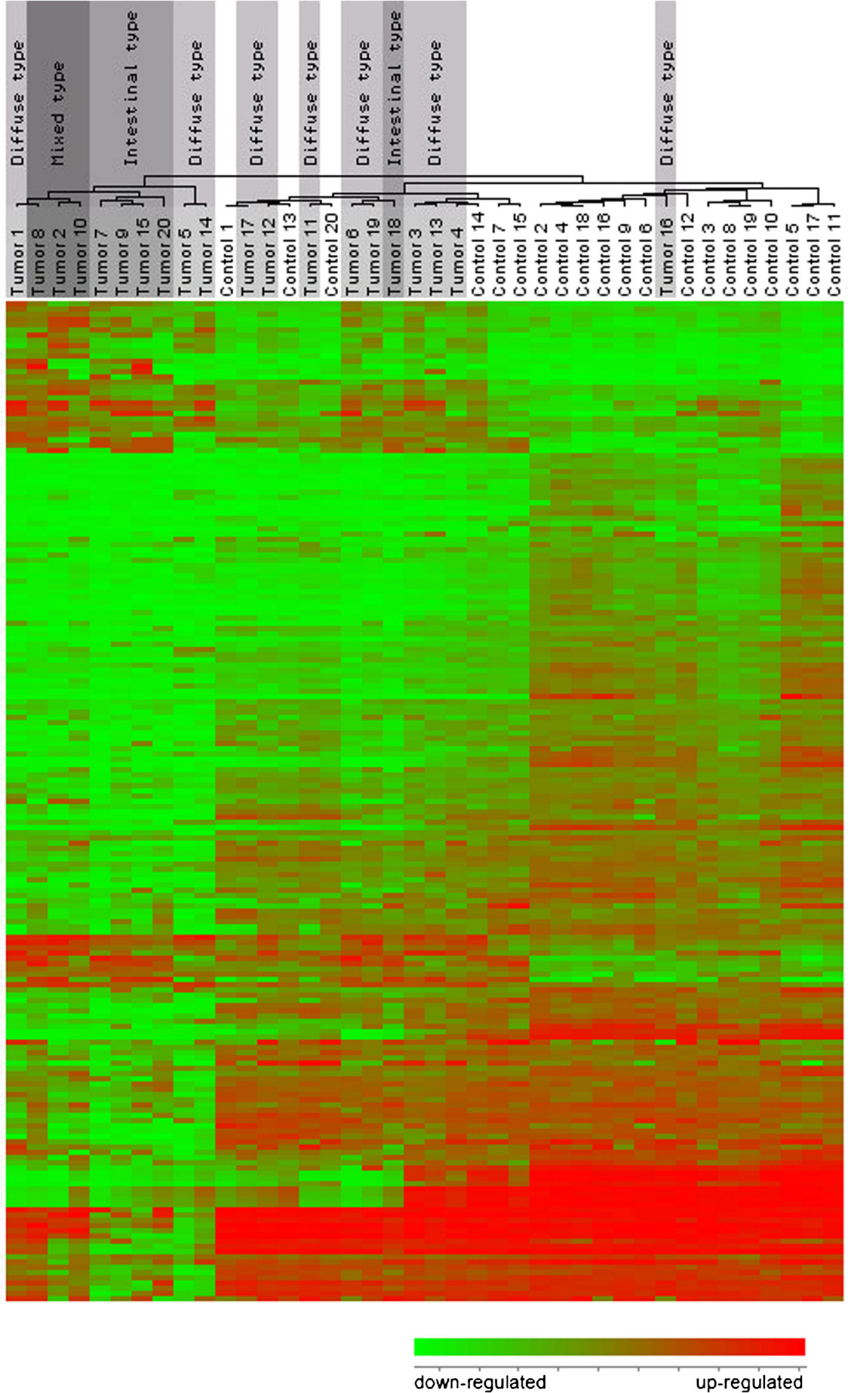
**Hierarchical clustering of the gene expression of 20 gastric tumors and control mucosa.** Whole genome expression of 20 tumor/control tissue pairs were filtered to produce a dataset containing the 130 most differentially regulated genes. Most the tumor samples clustered separate to the control samples. The diffuse type tumor samples are highlighted in light grey, the intestinal type in medium grey and the mixed type in dark grey to illustrate the subclustering of the three different histological cancer types.

Whole genome expression profiling of 20 matched gastric tumor samples was performed using cDNA microarrays. Rank product statistical testing [27] of the log_2_ fold change (FC) expression values of approximately 38000 genes on the microarray chip revealed 2297 genes that were significantly up-regulated and 2259 genes that were significantly down-regulated in the tumor tissue compared to matched normal mucosa (p < 0.01). The 130 filtered genes which were differentially regulated by an average FC > 1.5 are listed in Table 2, and constitute the dataset on which further analysis is performed. Of the most differentially regulated genes, 30 genes demonstrated up-regulation and 100 genes were down-regulated. *IL-8* was the single most up-regulated gene, up-regulated in 18 of 20 tissue pairs, with an average FC of 2.6 (Figure 1), followed by *COL1A1* and *CLDN1* (Figure 2). The most down-regulated gene was *PGA4*, being remarkably down-regulated in 18 of 20 tissue pairs, followed by *GIF* and *ATP4A*. Hierarchical clustering of the dataset (Figure 3) showed that the tumor and control tissues formed distinctly different gene expression clusters. Within the tumor cluster, the different histological categories diffuse, intestinal and mixed cancer formed almost exclusive individual clusters, demonstrating close genetic resemblance within each of the histological subsets. Among the control tissues, and among the *H. pylori* positive individuals, no particular clustering was seen.

**Table 2:** The most differentially regulated genes in gastric tumor vs control mucosa

**Up-regulated genes (n=30)**		**Down-regulated genes (n=100)**					
**Gene symbol**	**Average FC**	**Gene symbol**	**Average FC**	**Gene symbol**	**Average FC**	**Gene symbol**	**Average FC**
**IL-8**	**2.58**	PGA4	-5.58	MAL	-2.22	AKR7A3	-1.79
COL1A1	2.18	GIF	-5.48	SCNN1B	-2.22	**KIAA1324**	**-1.79**
**CLDN1**	**2.14**	ATP4A	-5.28	SOX21	-2.22	CCDC121	-1.78
SPP1	2.09	PGA3	-4.72	CAPN9	-2.21	FBP2	-1.76
CLDN2	2.09	ATP4B	-4.71	AGXT2L1	-2.20	FCGBP	-1.75
CEACAM6	2.09	PGA5	-4.34	HDC	-2.18	ORM2	-1.75
SERPINB5	2.06	LIPF	-3.91	GSTA1	-2.18	FAM3B	-1.73
**KRT17**	**2.00**	CPA2	-3.78	KLK11	-2.12	TRIM50	-1.73
H19	1.94	GHRL	-3.75	APLP1	-2.12	DUOX1	-1.72
**CLDN7**	**1.93**	GKN2	-3.26	MT1H	-2.09	RAP1GAP	-1.70
TFF3	1.92	KCNE2	-3.19	ADH1C	-2.09	EEF1A2	-1.70
OLFM4	1.91	SST	-3.12	DPCR1	-2.06	ANGPTL3	-1.70
THBS2	1.91	CHGA	-3.02	AKR1B10	-2.03	B3GAT1	-1.69
PI3	1.90	PSCA	-3.00	MT1G	-2.03	C6ORF105	-1.68
SULF1	1.89	CHIA	-2.88	CKB	-2.01	FGG	-1.68
BGN	1.82	GKN1	-2.88	SH3GL2	-1.99	**ADA**	**-1.65**
KRT6B	1.80	KCNJ16	-2.82	REP15	-1.97	C6ORF58	-1.63
THY1	1.72	GC	-2.66	CKM	-1.95	ZNF533	-1.60
MMP11	1.70	CLIC6	-2.65	FGA	-1.95	RPESP	-1.59
KLK6	1.67	SOSTDC1	-2.53	SLC9A4	-1.92	MT1F	-1.58
SERPINA3	1.65	ESRRG	-2.52	MFSD4	-1.92	PNPLA7	-1.57
FNDC1	1.64	CCKBR	-2.51	ALDOB	-1.89	FUT9	-1.57
COL1A2	1.63	TMED6	-2.44	SCNN1G	-1.87	RPRM	-1.56
CST1	1.63	MT1M	-2.44	IRX2	-1.87	GUCA2B	-1.56
FAP	1.60	**GPER**	**-2.43**	SLC26A9	-1.87	TCN1	-1.55
COL6A3	1.60	CKMT2	-2.36	CLCNKA	-1.87	PKIB	-1.55
SFRP4	1.56	VSIG2	-2.36	CAPN13	-1.86	**SLC9A2**	**-1.55**
TMEM158	1.53	FLJ42875	-2.33	TTR	-1.86	HOMER2	-1.53
**MMP7**	**1.50**	CXCL17	-2.32	GSTA2	-1.85	AKR1C4	-1.50
MMP10	1.50	CA9	-2.32	NKX6-2	-1.83	REG3A	-1.50
		AKR1C2	-2.27	CA2	-1.83	PI16	-1.50
		ALDH3A1	-2.24	FOLR1	-1.82	MAP7D2	-1.50
		SCGB2A1	-2.23	RDH12	-1.81		
		AQP4	-2.24	IRX3	-1.80		

Differentially regulated genes with average log2FC of > 1.5 (n = 130). extracted from whole genome expression. Average log2FC levels corresponding to each gene are listed. Nine genes. shown in bold type. demonstrated similar regulation in both the current study and in a previous study where gastric epithelial cells were exposed to *H. pylori*[17].

Genes from the current dataset were cross-matched against the most differentially regulated genes identified in our previous study, where gastric epithelial cells were exposed to *H. pylori* for 24 hours *in vitro*[17]. Both *H. pylori*-exposed gastric epithelial cells and the tumor biopsies demonstrated significant up-regulation of five common genes (*IL-8, CLDN1, KRT17, CLDN7* and *MMP7*) and down-regulation of four common genes (*GPER, KIAA1324, ADA* and *SLC9A2*).

### Gene ontology

Next, the dataset of the 130 most differentially regulated genes was analyzed for functional annotation using GO terms (Table 3). Among the 30 up-regulated genes, cell-adhesion processes, and in particular calcium-independent cell-cell adhesion, were among the most highly enriched terms. Furthermore, synthetic processes like skin morphogenesis and blood vessel development, as well as both catabolic and synthetic collagen-related processes were among the significant terms identified. Only a smaller proportion of the down-regulated genes were mapped to specific ontologies compared to the up-regulated genes, where digestion and excretion were the most enriched terms. Several metabolic processes, pH regulation and cobalamin and ion transport were also significantly enriched GO terms amongst the down-regulated genes (Table 4).

**Table 3:** Gene ontology associations in up-regulated genes

**P-value**	**No of genes involved**	**% of genes involved**	**Gene ontology**	**GO:number**
0.00066	3	10.0	Calcium-independent cell-cell adhesion	GO:0016338
0.0007	2	6.67	Skin morphogenesis	GO:0043589
0.023	5	16.67	Cell adhesion	GO:0007155
0.023	2	6.67	Collagen catabolic process	GO:0030574
0.023	2	6.67	Collagen fibril organization	GO:0030199
0.027	2	6.67	Blood vessel development	GO:0001568
0.042	1	3.33	Copulation	GO:0007620
0.042	1	3.33	Regulation of retroviral genome replication	GO:0045870
0.042	1	3.33	Responsen to corticosteroid stimulus	GO:0031960
0.042	1	3.33	Tooth mineralization	GO:0034505

Significantly enriched Gene Ontology terms in the up-regulated genes from the dataset.

**Table 4:** Gene ontology associations in down-regulated genes

**P-value**	**No of genes involved**	**% of genes involved**	**Gene ontology**	**GO:number**
0.0	8	7.41	Digestion	GO:0007586
0.00011	5	4.63	Excretion	GO:0007588
0.0005	3	2.78	Creatine metabolic process	GO:0006600
0.0019	3	2.78	Cellular aldehyde metabolic process	GO:0006081
0.0043	3	2.78	Regulation of pH	GO:0006885
0.013	2	1.85	Cobalamin transport	GO:0015889
0.015	9	8.33	Ion transport	GO:0006811
0.015	2	1.85	Secretion	GO:0046903
0.015	2	1.85	Cobalt ion transport	GO:0006824
0.02013	2	1.85	Morphogenesis of an epithelium	GO:0002009

Significantly enriched Gene Ontology terms in the down-regulated genes from the dataset.

### KEGG cellular signaling pathways

The dataset was then analyzed for KEGG cellular signaling pathway associations. 11 of the 30 up-regulated genes were mapped to 8 significant KEGG pathways (p < 0.05). In particular the cell adhesion molecules (CAM) pathway and leukocyte transendothelial migration pathway were assigned a high impact factor, due to the strong up-regulation *of CLDN1, CLDN7*, and *THY1* genes, and the high relative impact of these genes on the CAM pathway. *IL-8, COL1A1, COL1A2, THBS2, SPP1, COL6A3* and *SFRP4* were mapped to several highly impacted pathways: leukocyte transendothelial migration, extracellular matrix receptor interaction, tight junction, epithelial cell signaling in *H. pylori* infection, TGFβ signaling pathway, toll-like receptor signaling and Wnt signaling (Table 5). None of the 100 down-regulated genes were mapped to any significant KEGG pathways.

**Table 5:** KEGG cellular signaling pathways

**Rank**	**Pathway name**	**IF**	**P-value**
1	Cell adhesion molecules (CAMs)	734.0	0.000129
2	Leukocyte transendothelial migration	672.1	0.000879
3	ECM-receptor interaction	10.9	0.00215
4	Tight junction	10.6	0.000296
5	Epithelial cell signaling in Helicobacter	7.2	0.006
6	TGF-beta signaling pathway	6.3	0.013
7	Focal adhesion	6.2	0.014
8	Calcium signaling pathway	5.1	0.035

Significantly enriched KEGG cellular signaling pathways in the dataset (p < 0.05). Impact Factor (IF) is used to rank the affected signaling pathways, based on the fold change, the number of the involved genes in the pathway, and amount of perturbation of downstream genes.

### Clinicopathological correlation

Among the total set of the 130 most differentially regulated genes, the FC expression levels of 20 genes showed significant correlation with post-operative survival, 8 genes correlated with histological type, 5 genes correlated with tumor size, and 1 gene correlated with lymph node stage (Additional file 1). Some genes showed correlation with more than one parameter. Because of the moderate sample size (n = 20), a significance level of p < 0.01 was chosen.

Cox multivariate analysis of the genes associated with post-operative survival demonstrated that a high *CLDN1* expression level was the only independent predictor gene of post-operative survival. *CLDN1* expression and the covariates tumor size, positive lymph node fraction, histological type, gender and age at surgery were entered into a linear regression model (Table 6), demonstrating that only *CLDN1* and positive lymph node fraction were significant predictors of post-operative survival. When all non-significant determinants where removed, there was a stronger negative correlation between *CLDN1* expression and post-operative survival (R = -0.7, p < 0.001). There were no significant association between *CLDN1* expression and Lauren classification, *H. pylori* infection, ethnicity, tumor size or metastatic lymph node status.

**Table 6:** Factors influencing survival following surgery for gastric cancer

**Co-variate**	**Correlation coefficient R**	**P-value**
*CLDN1* expression (log_2_ fold change)	-0.53	0.008
Lymph node fraction	-0.46	0.016
Tumor size	-0.27	0.186
Age at surgery	-0.13	0.491
Histological type (intestinal type)	-0.03	0.888
Male gender	-0.05	0.792

Linear regression analysis of multiple factors influencing post-operative survival in patients undergoing surgery for gastric cancer.

**Figure 4:**
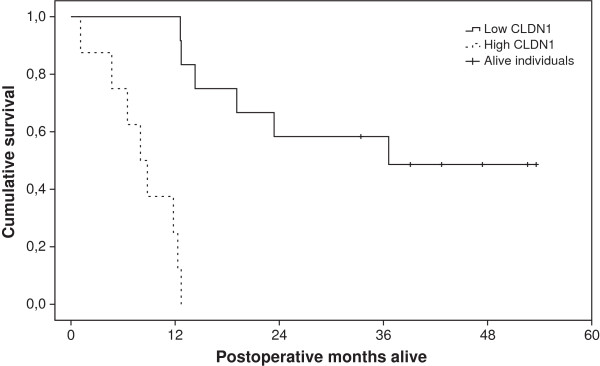
**Kaplan Meier survival plot of patients with resected gastric tumors.** The solid line represents below average *CLDN1*-expressing tumors (FC < 2.14) and the dotted line represents above average *CLDN1*-expressing tumors (FC > 2.14). 6 patients in the low *CLDN1*-expressing group were still alive at the end of the study period, as demonstrated by the solid line vertical tics.

To detect differences in post-operative survival between individuals with high and low *CLDN1*-expressing tumors, different cut-off levels were utilized to create high-expressing and low-expressing groups. Using the *CLDN1* FC mean (FC mean = 2.14) as the group divider, high- and low-expressing *CLDN1* patients demonstrated significantly different survival patterns (p < 0.001) as illustrated in the Kaplan-Meier plot in Figure 4.

### Histopathological features of adjacent non-cancerous mucosa

**Figure 5:**
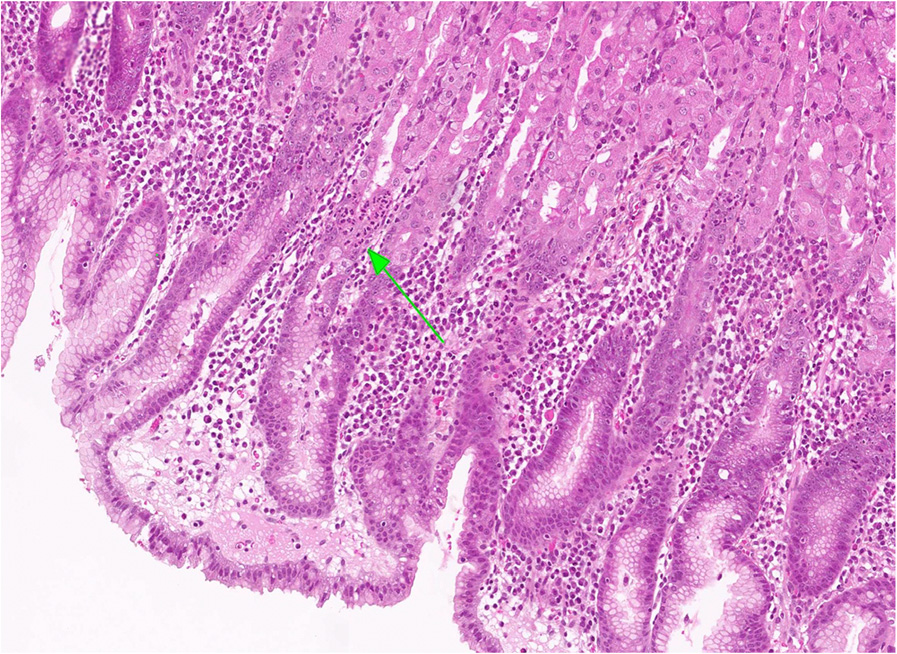
**Histological section of *H. pylori *induced non-atrophic chronic gastritis.** The arrow points out an area of active inflammation with characteristic granulocytic infiltration in the crypt epithelium.

**Figure 6:**
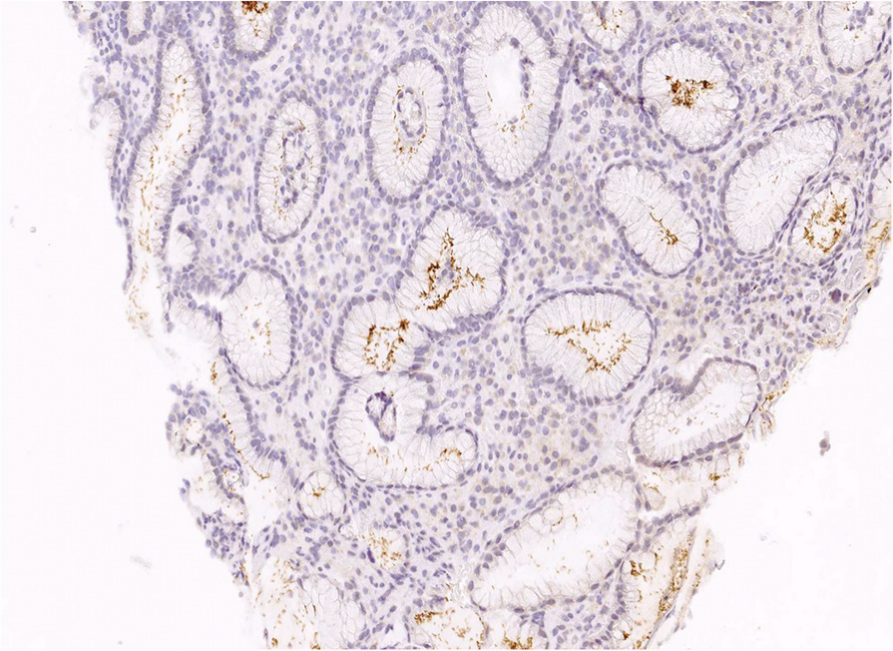
**Immunohistological evidence of *H. pylori. H. pylori *was discovered in 2 of the 20 mucosa samples.** The specimen from Figure 5 has been subjected to immunohistochemistry treatment by anti-*Helicobacter*-antibody, staining *H. pylori* brown.

Of the 20 matched mucosa specimens, 10 showed evidence of non-atrophic gastritis, and 10 demonstrated multifocal atrophic gastritis. Intestinal metaplasia was scored from 1–3 in the antrum and corpus areas of the samples. The intestinal type tumors were significantly associated with both atrophic gastritis and intestinal metaplasia (p < 0.001), whereas the diffuse type tumors were associated with non-atrophic gastritis (p < 0.001). Both histological and immunohistochemical evidence of *H. pylori* were demonstrated in the mucosa counterpart of 1 intestinal and 1 diffuse type cancers (Figures 5 and 6), both in caucasian patients. The other 18 specimens showed no evidence of *H. pylori*. There were no significant differences between types of cancers, types of gastritis, or the presence of *H. pylori* on the one hand, and the correlation with the gene expression of *CLDN1* or *IL-8*.

## Discussion

In this study we identified *CLDN1* as one of the most consistently up-regulated genes in GC and a strong correlation between up-regulation of *CLDN1* and reduced survival in 20 patients with gastric adenocarcinomas. This correlation is even stronger when adjusted for other parameters such as lymph node stage, tumor size and histological type. Our clinical sample size is small, but the results are consistent.

Claudins are proteins involved in cellular tight junctions and are important for the maintenance of normal epithelium, in particular barrier formation, cell polarity and signal transduction. Dysregulation of these genes have been identified in many different cancers. Based on tumor biology, down-regulation of *CLDN1* would result in destruction of tight junctions and loss of cell-to-cell adhesion causing tumor progression [33], however the clinical significance in gastric carcinogenesis is more complex. There is evidence that several of the claudins, *CLDN1* included, show increasing levels as gastric epithelium progresses to intestinal metaplasia and early gastric carcinoma [34]. *CLDN1* might influence intracellular signalling, demonstrated by Liu et al. who showed that elevated expression of CLDN1 in breast cancer cells contributed to an anti-apoptotic effect through two mechanisms: inhibition of caspase-8 cleavage, and activation of the Wnt/β-catenin signal pathway [35]. CLDN1 has been identified within the nucleus of gastric cancer AGS cells *in vitro*, suggesting a regulatory role of CLDN1 on cell proliferation, migration and invasiveness at a nuclear level [36]. Some studies on ovarian and colon cancer report a role of CLDN1 on metastatic processes through activation of metalloproteinases, reducing apoptosis and increasing migration [36]. Although there are several papers to support an oncogenic role of CLDN1 in gastric cancer, two studies nevertheless showed reduced CLDN1 staining in metastatic compared to non-metastatic gastric cancer [33], and increased tumorgenicity in *CLDN1* negative gastric epithelial cells [37], contrasting our findings.

The expression pattern of *CLDN1* differs not only between different stages of carcinogenesis, but also between histological subtypes and between regions of the gastrointestinal tract. Resnick et al. demonstrated increased staining of CLDN1 protein in intestinal compared to diffuse type gastric cancer [38]. In contrast, Jung et al. demonstrated significantly lower CLDN1 expression in intestinal compared to diffuse type [33]. Neither studies found any correlation between CLDN1 and prognosis. Wu et al. demonstrated positive correlation between CLDN1 expression and invasiveness and metastasis in gastric tumors using immunohistochemistry [39]. In two studies on colorectal cancer, low expression of CLDN1 was a predictor of poor prognosis [40,41], however an association between high *CLDN1* expression and depth of tumor invasion was also noted [41]. In summary, the role of CLDN1 in cancer progression and prognosis is far from clear.

Our data demonstrate a marked increase in *CLDN1* expression in 19 of 20 tumors compared to normal tissue, with a significant and independent relationship between high *CLDN1* expressing tumors and reduced postoperative survival. We found no statistically significant difference between *CLDN1* expression and histological subtypes, for that our sample number is insufficient. Nevertheless, tumors with high *CLDN1* expression (FC > 2.14) showed an extremely poor prognosis as there were no patients alive at 450 days following curatively intended surgery in this group. In the low-expressing *CLDN1* group (FC < 2.14), patients showed significantly longer post-operative survival, and 50% of the patients were still alive at the end of the study. We have reported total mortality and not cancer specific mortality. Our sample number is small, and a much larger study would be required to reveal statistically significant correlation between *CLDN1* expression and sample subgroups, such as the histological subtypes, different tumor stages and *H. pylori* status. The role of *CLDN1* in gastrointestinal cancer is controversial, but it seems convincing that the high *CLDN1* gene expression conferred a very unfavorable prognosis in our study population. Moreover*, CLDN1* was also one of the most significantly up-regulated genes in the previously studied *H. pylori*-exposed gastric epithelial cells [17], suggesting a possible causal relationship between chronic *H. pylori* exposure and *CLDN1* up-regulation in gastric mucosa.

Other claudins that were up-regulated in the tumor samples were *CLDN2* and *CLDN7,* and these genes might also be implicated in gastric carcinogenesis [36,42-48]. *CLDN7* was also among the most significantly up-regulated gene in the *H. pylori* exposed gastric epithelial cells [17], suggesting a role of this bactierium in the regulation of this gene.

The most consistently increased gene in the study was *IL-8*, up-regulated in the tumor in 18 of 20 tissue pairs*. IL-8* is one of the major mediators of inflammation and a powerful chemokine that targets neutrophils and lymphocytes through the receptors CXCR1 and CXCR2. *IL-8* is paramount in the acute inflammatory response to *H. pylori* infection and is also increased in chronic gastritis [49]. The increased *IL-8* expression in the tumor samples may represent intratumoral inflammation as a normal reaction to an abnormal environment. However, the absence of other acute or chronic inflammatory genes suggests that the up-regulated *IL-8* in the tumors can not be entirely explained by an inflammatory process alone. Hence, the role of *IL-8* in the gastric cancer is not clear. First, persistent and chronic inflammation in the stomach is associated with an enhanced production of several pro-inflammatory cytokines including *IL-8*[50] which increases apoptosis, hyperproliferation and production of reactive oxygen and nitrogen species causing DNA damage and mutations. Second, increased vascularization is one of the hallmarks of malignant transformation, and *IL-8* may serve an important role in this process. Several authors have demonstrated promotion of angiogenesis in tissue exposed to IL-8 protein [51-53]. A plausible causal role of IL-8 in the growth and vascularization of gastric cancer has also been shown in the work of Kitadai et al., where IL-8 transfected cells that were injected into the gastric wall of mice, rapidly produced growth of highly vascularized tumors [54]. Interestingly, we also found significantly increased and coordinated up-regulation of *COL1A1* and *COL1A2* in the tumor tissue, both of which are important in blood vessel development. Two authors recently found an association between IL-8 and adhesion, migration, and invasion in gastric cancer cells [55,56]. Targeting of the IL-8 receptor CXCR2 has been suggested as a novel cancer treatment in several studies [53,55-57].

In our previous study we identified *IL-8* as the single most up-regulated gene in the acute response of gastric epithelial cells exposed to *H. pylori in vitro*[17]. *IL-8* is also up-regulated in the pre-malignant stages of gastric cancer, such as chronic gastritis [58] and intestinal metaplasia [59]. With *IL-8* being currently demonstrated as the single most up-regulated gene in surgically resected GC tumors, the up-regulation of this gene throughout gastric cancer progression may constitute an early and important event in the disease, initiated and maintained by *H. pylori* infection.

The causal relationship between *H. pylori* colonization of the stomach and GC has been widely accepted [60]. In this study only 2 of the 20 gastric tumors showed active *H. pylori* infection at the time of surgery, 1 intestinal and 1 diffuse type cancer. While more than 90% of the GC reported in Asian countries are considered attributable to this bacterium [61], our findings might indicate a lesser role of *H. pylori* in gastric cancer in a country like Norway. However, GC tissue is frequently *H. pylori* negative, due to the mucosal atrophy caused by the bacteria itself. While *H. pylori* colonization drives forward the progression of mucosal atrophy and intestinal metaplasia, this process paradoxically also slowly eradicates the same bacteria from the gastric mucosa which causes a decrease in active inflammation [62].

In the current study, we demonstrated up-regulation of several matrix metalloproteinases (MMPs); and *KRT17* in the tumor of almost all the tissue pairs. MMPs participate in the degradation of extracellular matrix and the regulation of tumor growth and angiogenesis, and are important in the detachment of malignant cells from adjacent tissue to attain metastatic ability. Keratin 17 has been shown to be over-expressed in several adenocarcinomas including GC, and an association with aggressive tumor behaviour, local invasion, metastasis as well as treatment responsiveness has also been suggested [63-65]. We found no assiciation between MMPS and *KRT17* and clinicopathological parameters. However, other studies have demonstrated an association with MMPs and advanced tumor stage, high grade tumors and metastasis, as well as a role for MMP11 as a serum marker in GC disease [66-69]. *MMP7* and *KRT17* were also among the most up-regulated genes our previous study of gastric epithelial cells exposed to *H. pylori*[17], which raises the possibility of a role of *H. pylori* also in the regulation of these genes.

In hiarchical clustering of the samples, the tumor and the control tissues clustered separately. Normal mucosa tissue bore a closer biological resemblance to normal mucosa from the other individuals, than to the tumor counterpart from its own stomach. In similar fashion, most tumor samples clustered together, demonstrating common genetic features between the tumor specimens. In addition, there was a large horizontal distance between the tumor specimen and the normal specimen within each tissue pair, illustrating that a significant shift in gene expression has occurred during the progression from normal mucosa to cancer within the same stomach. Furthermore, the mixed type and the intestinal type of cancers formed two almost exclusive clusters compared to the diffuse type cancers, indicating that each of the histological types have distinct gene expression profiles. Surprisingly, the mixed type cluster showed the greatest difference in gene expression compared to the normal tissue, indicating that the biology of this type of GC has removed itself the most from the original naive tissue. The results are interesting, but the sample number is too small to draw any conclusions.

We performed GO analyses to cluster the most differentially regulated genes according to biological function. Several terms relating to cell adhesion were enriched by genes up-regulated in the tumor samples. KEGG pathway analysis also showed that a number of pathways regulating cell attachment were significantly affected, in agreement with the GO analysis. Disruption of primary cell attachments, and secondly, cell adhesion to distant sites, are two fundamental steps in the ability of tumor cells to develop metastatic disease. Blood vessel development is essential for the tumor’s ability to survive and metastasize to distant sites. This gain of abilities seems to come at the cost of loss of features of differentiated intestinal tissue, such as digestive and excretive processes, which were associated with down-regulated genes. While several of the highly up-regulated genes were mapped to important pathways, none of the 100 down-regulated genes were involved in any significant pathways, indicating that it is the net gain of oncological function that translates into tumor growth and malignant behavior, rather than the loss of tumor suppression.

## Conclusion

In our study, *CLDN1* was not only highly up-regulated in the gastric cancer samples, but *CLDN1* expression was also independently associated with a very poor post-operative prognosis. *IL-8* was the single most up-regulated gene in the study, and we have shed new light on the role of both genes in gastric carcinogenesis. *IL-8* and *CLDN1* may represent important links between GC and the gene response seen in acute *H. pylori* infection of gastric epithelial cells. Intestinal, diffuse and mixed type of gastric cancer formed separate gene expression clusters, but also showed many genetic similarities. Functional analysis demonstrated that several cellular pathways regulating cell attachment were affected in the tumor tissue.

## Competing interests

All authors declare that they have no competing interests.

## Authors’ contributions

LLE, IRKB and GB participated in the design of the study. LLE obtained all biopsies and performed mRNA isolation with YE. GPB performed the histological and immunohistological examination. LLE carried out the microarray data analysis and wrote the main manuscript, with contributions from the other authors. All authors read and approved the final manuscript.

## Pre-publication history

The pre-publication history for this paper can be accessed here:

http://www.biomedcentral.com/1471-2407/13/586/prepub

## Supplementary Material

Additional file 1 **Correlation between differentially regulated tumor genes and clinicopathological parametres.** Correlation coefficients between the most differentially regulated genes in tumor tissue and clinicopathological parameters (p < 0.01, n = 31). Only genes with at least one significant clinicopathological correlation is shown, extracted from the filtered dataset of the 130 most differentially regulated genes. The 99 other genes omitted from the list did not show correlation with any clinicopathological parameter. Empty table cells denote no significant correlation. Pearson and Spearman coefficients listed. The file is in Adobe PDF format, best viewed in Adobe Acrobat Reader. Click here for file
