# Supplementary material for: Up-regulation of CLDN1 in gastric cancer is correlated with reduced survival
Source: BMC Cancer. 2013 Dec 10;13:586. doi: 10.1186/1471-2407-13-586 (PMC4029627; doi:10.1186/1471-2407-13-586)
Supplement: Additional file 1 — Correlation between differentially regulated tumor genes and clinicopathological parametres. Correlation coefficients between the most differentially regulated genes in tumor tissue and clinicopathological parameters (p < 0.01, n = 31). Only genes with at least one significant clinicopathological correlation is shown, extracted from the filtered dataset of the 130 most differentially regulated genes. The 99 other genes omitted from the list did not show correlation with any clinicopathological parameter. Empty table cells denote no significant correlation. Pearson and Spearman coefficients listed. The file is in Adobe PDF format, best viewed in Adobe Acrobat Reader. [file 1471-2407-13-586-S1.pdf]

Correlation coefficients of the most differentially regulated tumor genes and clinicopathological parametres

Gene symbol	Postoperative survival		Tumor size		Intestinal subtype		Lymph node metastasis	
	Pearson	Spearman	Pearson	Spearman	Pearson	Spearman	Pearson	Spearman
ADA	-0.633	-0.604						
ADH1C				-0.578				
AGXT2l1	-0.684	-0.595						
ANGPTL3	-0.702	-0.656						
APLP1	-0.740	-0.635						
AQP4	-0.692	-0.582						
B3GAT1	-0.724	-0.721						
C6ORF58				-0.569				
CAPN9						-0.651		
CCDC121		-0.563						
CHIA	-0.608	-0.565	0.566					
CKM	-0.626	-0.604						
CLCNKA	-0.673	-0.611						
DPCR1						-0.572		
CLDN1	-0.58	-0.693						
FGA	-0.626	-0.677						
FLJ42875	-0.592							
GKN1					-0.573	-0.606		
KCNJ16		-0.580						
KLK6					0.575	0.574		
KLK11		-0.709						
MFSD4	-0.652							
RAP1GAP								0.574
REP15						-0.577		
RPRM	-0.680	-0.719	0.579					
SCGB2A1					-0.587			
SH3GL2	-0.732	-0.629	0.572					
SOSTDC1					-0.571	-0.594		
SOX21					-0.606	-0.596		
TMED6	-0.578	-0.616						
TRIM50	-0.708	-0.693						
